# Editorial

**Published:** 1872-12

**Authors:** 


### Editorial.

***Last No. of the Volume.***

Subscribers will take notice that this is the final No. for 1872. Prompt remittance for 1873 is desirable, that our publishers may know how many copies to print. The very expensive style in which the JOURNAL is sent out to its readers, renders it necessary that no more extra copies should be issued than are absolutely required. Payment in advance will continue to be the invariable rule. No pains will be spared on the part of the publishers to keep up the high standard of typographical excellence which under their management has heretofore characterized the JOURNAL.

***Contributors***

Are cordially thanked for the variety and value of the articles which they have afforded us the year now concluding, and continuance of their favors is both earnestly solicited and confidently expected. We shall welcome new recruits with great pleasure.

***The Editors***

Will do their best to make the JOURNAL a vehicle for the most important and interesting productions of medical thought and observation, wheresoever they may be secured. The mere writing of "editorials" is the smallest part of an editor's duty—although some have the idea that that is the only thing to be done. The medical journal should be the reflex of the advanced opinions, and the chronicle of the discoveries and improvements of the profession. The advancement of personal interests, the dissemination of pet notions, the cultivation of professional polemics, or even denunciation of quacks, although well enough in their place, should not, in our opinion, be allowed to usurp space and time that can be devoted to higher uses.

Freedom of inquiry never damages when clear heads interrogate. Freedom of expression never injures where ordinary courtesy governs the individual. It is possible for the scientific medical journal to powerfully influence professional thought and observation without sermonizing on the matter.

In the future, as in the past, this JOURNAL will seek to promote the highest interests of the profession by illustrating what it thinks and does, and not merely clamors about and assumes.

The Index of the volume that closes with this No. will give "in a nutshell" an idea of what the year has accomplished. Favor us with your subscriptions and your contributions, and 1873 shall be as 1872, "but much more abundant."

***Clinical Charts.***

W.W. Keen, M.D., of Philadelphia, has issued, through Turner Hamilton, Publisher, a series of diagrams to facilitate the keeping of clinical notes. By their use the observations made are indicated to the eye at once, avoiding long verbal descriptions. The physical signs, locations of diseases, measurements, etc., etc., can be noted in a moment. A final chart enables a record of the pulse, temperature and respirations to be made and appreciated *tuto, cito et jucunde.* Address the publisher as above, No. 106 South Tenth Street, Philadelphia.

***Lindsay & Blakiston***

Have issued another edition of Aitken's Practice, and although large additions have been made, they have presented it in a more compact form than previous editions. We take pleasure in calling attention to their new and revised catalogue of publications, wherein we find noted many new and valuable books. They will send the new catalogue free to any person desiring it. Address them at Philadelphia.

***Epizootic.***

The horse influenza has nearly disappeared from this city, but is still ravaging many parts of the country. We hope that some of our friends will furnish for the JOURNAL a complete and scientific history of this enormous nuisance—for thus it has really proved, especially to physicians in this city of magnificent distances. *Apropos* to the epizootic, we are gratified in being able to announce that measures are being taken to secure a higher grade of professional care and management of that noblest of quadrupeds, the horse. The epizootic will not have been without its use if it should result in the establishment of a veterinary college of superior merit in this city. The poor horse has been a long-suffering animal, that will hereafter be more highly prized and better cared for than ever heretofore. The Illinois Humane Society, through their efficient agent, Dr. Charles T. Zaremba, are giving the subject their immediate attention.

***Dangerous Formula.***

Fred. Clemner, M.D., of Waterloo, N. Y., calls the attention of readers to a formula given by a correspondent of the JOURNAL in June last, embracing in one prescription, Iod. Potassii, Potassæ Chlorat. and Potassæ Carb. It will be noticed that the chemical changes ensuing would give compounds of a highly irritant if not poisonous nature. *Verb. Sap.*

***Vick's Floral Guide for 1873.***

The Guide is now published quarterly; 25 cents pays for the year, four numbers, which is not half the cost. Those who afterwards send money to the amount of one dollar or more for seeds may also order 25 cents worth extra—the price paid for the Guide. The January number is beautiful, giving plans for making rural homes, designs for dining table decorations, window gardens, etc., and containing a mass of information invaluable to the lover of flowers. One hundred and fifty pages, on fine tinted paper, some five hundred engravings, and a superb colored plate and chromo cover. The first edition of two hundred thousand just printed in English and German, and ready to send out. JAMES VICK, Rochester, N. Y.

---

***Arsenic in Dyspepsia.***

Dr. J. C. Thorowgood, in the *Practitioner*, speaks highly of the action of arsenic in many diseases of the stomach. He has found that one-drop doses of Fowler's solution in half an ounce of infusion of columbo had the effect, in a case he treated, of allaying the pain, stopping the vomiting of food, and enabling the patient to eat and digest small quantities of mutton. He states that the usual irritable tongue, with projecting papillæ and yellow or gray fur, indicate arsenic. The more purely local the gastric symptoms, the better is the chance of arsenic doing good. Where there is much general exhaustion of the system, with disordered urine or hepatic congestion, it does not promise much.—*The Georgia Medical Companion.*

